# Mini Profile of Potential Anticancer Properties of Propofol

**DOI:** 10.1371/journal.pone.0114440

**Published:** 2014-12-11

**Authors:** Jing Song, Yenji Shen, Jing Zhang, Qingquan Lian

**Affiliations:** 1 Department of Anesthesiology, Montefiore Medical Center, New York, New York, United States of America; 2 Department of Anesthesiology, the Second Affiliated Hospital & Yuying Children's Hospital of Wenzhou Medical University, Wenzhou, Zhejiang, China; Massachusetts General Hospital, United States of America

## Abstract

**Background:**

Propofol (2, 6-diisopropylphenol) is an intravenous sedative-hypnotic agent administered to induce and maintain anesthesia. It has been recently revealed that propofol has anticancer properties including direct and indirect suppression of the viability and proliferation of cancer cells by promoting apoptosis in some cancer cell lines.

**Methodology/Principal Findings:**

This study aimed to establish a profile to quantitatively and functionally evaluate the anticancer properties of propofol in three cancer cell lines: non-small cell lung carcinoma cell line A549, human colon carcinoma cell line LoVo, and human breast cancer cell line SK-BR-3. We demonstrated that the expression level of caspase-3, an apoptosis biomarker, significantly increased in a dose-dependent manner after 24-h stimulation with 100 µM propofol in A549 cells, and slightly increased in LoVo cells. However, there was no change in caspase-3 expression in SK-BR-3 cells. High caspase-3 expression in A549 cells may be modulated by the ERK1/2 pathway because phosphorylated ERK1/2 dramatically reduced after propofol treatment. BAX, a major protein that promotes apoptosis in the regulation phase, was highly expressed in A549 cells after treatment with 25 µM propofol. Apoptosis induced by propofol may be associated with cancer cells carrying Kras mutations.

**Conclusions/Significance:**

Our results suggest that the anti-cancer effects of propofol, which are consistent with those of previous studies, are likely associated with the Kras mutation status. Only Kras mutation in Codon 12 instead of other Kras status has been demonstrated to play an important role in sensitizing the propofol-induced apoptosis in cancer cell lines from our study. These findings may enable us a detailed investigation of propofol/Kras-mediated cancer cell apoptosis in the future.

## Introduction

Propofol (2, 6-diisopropylphenol) is an intravenous sedative-hypnotic agent with characteristics of smooth induction and rapid recovery from anesthesia. Propofol has been shown to possess anti-cancer properties [1], [2], [3] which is in contrast with some of the other anesthetics that appear to suppress immunity and assist cancer growth [4], [5], [6], [7].

Numerous studies have confirmed that both cell-mediated immunity and humoral immunity play an important role in determining the spread of cancer cells and micro metastasis [8]. Propofol reportedly increases the expression of CD28 and the ratio of IFN-γ/IL-4 in patients with lung cancer undergoing pulmonary lobectomy, which indicated that propofol may initiate the activation of T-helper cells and promote the differentiation of T-helper 1 cells [6]. It has been suggested that clinically relevant concentrations of propofol not only inhibit in vitro human colon cancer cell invasion and metastasis via reduction of matrix metalloproteinases [9], it promotes apoptosis and directly suppresses the viability and proliferation of human promyelocytic leukemia cells in vitro as well [10]. Also, there is evidence suggesting the role of propofol as a weaker modulator of apopotosis in certain closely related human promyelocytic leukemia cells [10]. Different cell types exhibited different and diverse levels of sensitivity to apoptosis after propofol stimulation [10]. Furthermore, researchers detected that propofol, when conjugated with omega-3 polyunsaturated long-chain fatty acids, had a profound effect on inducing apoptosis with a significant increase in caspase-3 levels in breast cancer cell MDA–MB-231 [7].

Propofol may also be associated with the deactivation of the EGFR signaling pathway [9], [11], [12]. This pathway is involved in the regulation of cancer invasion as well as in cancer cell proliferation. Kras, a well-known oncogene in the EGFR signaling pathway, plays a critical role in the fate of tumor cell survival depending on its genotype. Mutations in specific codons of the KRAS oncogene result in different clinical therapeutic responses [13].

Although propofol induces apoptosis and inhibits the invasion of cancer cells both in vitro and in vivo via different molecular mechanisms [5], [9], we focused on the anti-cancer properties of propofol that are regulated via EGFR signaling pathway. The aim of this study is to establish a mini profile with three different cancer lines (the Kras mutant cell lines: non-small cell lung carcinoma A549 and human colon carcinoma LoVo, and the Kras wild-type cell line: human breast cancer SK-BR-3) to quantitatively and functionally evaluate the apoptotic effect with different doses of propofol, and thereby determine whether propofol might be advantageous as an anesthetic for surgeries of certain cancers.

## Materials and Methods

The study was approved by the Medical Research Committee in the Wenzhou Medical College, China.

### Cell lines

A549 lung cancer cells, LoVo colon cancer cells, and SK-BR-3 breast cancer cells were obtained from the Shanghai Institute of Cell Biology, Chinese Academy of Sciences. Cells were cultured in RPMI 1640 media (Sigma, St. Louis, USA) supplemented with 10% fetal bovine serum, 100 U/ml of penicillin and 100 µg/ml of streptomycin at 37°C in a humidified incubator with 5% CO_2_.

### Reagents and Chemicals

Propofol (Aldrich, Milwaukee, WI) was diluted in dimethyl sulfoxide (DMSO) for in vitro assays. All primary antibodies (caspase-3 (H-277), Bax (N-20), and p-ERK 1/2 (Thr 202) for High Content Analysis (HCA) and Western blot analysis were from Santa Cruz biotechnology (Santa Cruz, CA). Alexa Fluro 488 F (ab)'2 fragment of Goat anti Rabbit secondary antibody and Hoechst 33342 were from Invitrogen (USA).

**High Content Analysis (HCA)** A549, LoVo, and SK-BR-3 cells were grown in 96 well plates (BD Falcon Optilux black 96 well plate) with various concentrations of propofol (25 µM, 50 µM, and 100 µM) for 6 hours (2,000 cells per well) and 24 hours (1,000 cells per well). The cells were then fixed in 4% paraformaldehyde. The nuclei were stained with 20 µg/ml of Hoechst 33342 (Invitrogen, USA) for 30 minutes at room temperature. The caspase-3 or BAX was stained as follows: the cells were washed with cold PBS and permeabilized with 0.5% Triton ×100 for 10 minutes at room temperature, then incubated with 2% goat serum albumin for 30 minutes to block the background. Further, the cells were incubated with primary antibodies against either caspase-3 or BAX, with the dilutions as suggested by the producer, overnight at 4°C. Finally, the cells were washed with 1×PBS and incubated with a secondary antibody (Alexa Fluor 488 Goat Anti-Rabbit IgG (H+L), 1∶500dilution). Immunoreactivity of each sample was observed and analyzed with High Content Analysis (Celomic Array Scan VTI 636, Thermo Scientific). The procedure described above follows the manufacturer's Target Activation BioApplication Guide.

### Western blot analysis

A549, and LoVo cells were plated in six well plates with or without propofol stimulation at different times (5 h and 1 h). The cells were washed with cold PBS, then harvested in RIPA buffer. Protein concentrations were determined with a BCA Protein Assay Kit (Beyotime, China). Equal protein amounts were run on SDS-PAGE gels and transferred to nitrocellulose membranes. The membranes were blocked for 1 hour at room temperature in PBS with 0.1% Tween-20 containing 5% nonfat milk or 5% BSA, then probed with primary antibodies against phosphorylated ERK1/2. After incubation with peroxidase-conjugated anti-rabbit IgG, the signal was revealed with enhanced chemiluminescence (ECL) reagent (Pierce, USA).

### Bioinformatics Data Search

All oncogene data is referenced via the bioinformatics data search from the Cancer Cell Line Encyclopedia (CCLE) open resource at http://www.broadinstitute.org/ccle/home.

### Statistical analysis

One-way ANOVA by GraphPad Prism 6 was used to evaluate statistical differences among experimental groups followed by Dunnett's test. A value of p<0.05 was considered significant with the confidence interval (C.I.) set at 95%. Result values are shown here as the mean ± SEM from three independent experiments.

## Results

### Caspase-3 Functional Expression and Quantification with HCA

**Figure 1:**
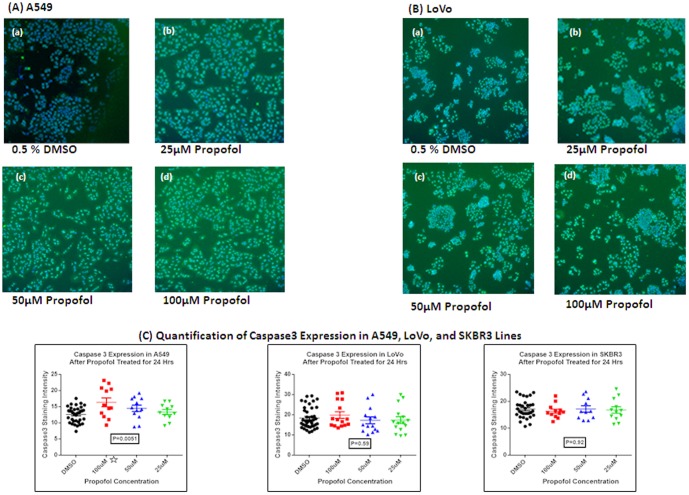
High Content Analysis of caspase 3 expression in three cell lines. Propofol treatment induces apoptosis in A549 cells significantly, slightly induces apoptosis in LoVo cells, and no change in SKBR3 cells. (A) High content analysis of caspase-3 expression in A549 cells treated with the indicated propofol concentrations for 24 hours. Nuclei were stained with Hoechst 33342 shown in blue color. Caspase-3- positive stained cell are shown in green color. Images were acquired at 5× magnification lens. (a) A549 cells treated with 0.5% DMSO for 24 hours. (b) A549 cells treated with 25 µM of propofol in 0.5% DMSO for 24 hours. (c) A549 cells treated with 50 µM of propofol in 0.5% DMSO for 24 hours. (d) A549 cells treated with 100 µM of propofol in 0.5% DMSO for 24 hours. These results are representative of three independent experiments. (B) High Content analysis of caspase-3 expression in LoVo cells treated with indicated propofol concentrations for 24 hour. Nuclei were stained with Hoechst 33342 shown in blue color. Caspase-3 positive stained cells are shown in green color. Images were acquired at 5× magnification lens. (a) LoVo cells treated with 0.5% DMSO for 24 hours. (b) LoVo cells treated with 25 µM of propofol made with 0.5% DMSO for 24 hour. (c) LoVo cells treated with 50 µM of propofol made with 0.5% DMSO for 24 hour. (d) LoVo cells treated with 100 µM of propofol made with 0.5% DMSO for 24 hour. These results are representative of three independent experiments. (C) Charts indicating the caspase-3 expression in A549 cells (a), LoVo cells (b), and SKBR3 cells (c) with the administration of propofol for 24 hours. There is no change after 6 hour treatment with propofol for any of these three cell lines tested here (Data not shown). Each value was determined with Target Activation BioApplication Guide suggested by manufactory (Thermo Scientific), and normalized to the expression of cpaspase 3 with 0.5% DMSO incubation. The results were evaluated by ANOVA test. A star (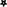) indicates statistically significant with the P<0.05.

In caspase-mediated apoptotic pathways, there is an activation of a series of caspase family proteases. Caspase 3, a key destructive enzyme, is involved in both the death-receptor-mediated and mitochondria- dependent pathway [14]. To determine the effects of propofol on the induction of apoptosis for these three cell lines (A549, LoVo, and SKBR3), caspase-3 was selected as an apoptotic marker in our study. As shown in Fig. 1, caspase-3 was significantly more highly expressed in the A549 cell after 24-hours of 100 µM of propofol treatment, in a dose-dependent manner, compared with controls. In the LoVo cell, there was a trend toward high expression for caspase-3 after 24-hours treatment with 100 µM of propofol. Propofol had no such effect on SKBR3 cells.

### BAX Functional Expression and Quantification with HCA

BAX as one of the Bcl 2 family members involved in the mitochondria-dependent pathway of apoptosis is also a commonly used as a pro-apoptotic biomarker [15], [16].

**Figure 2:**
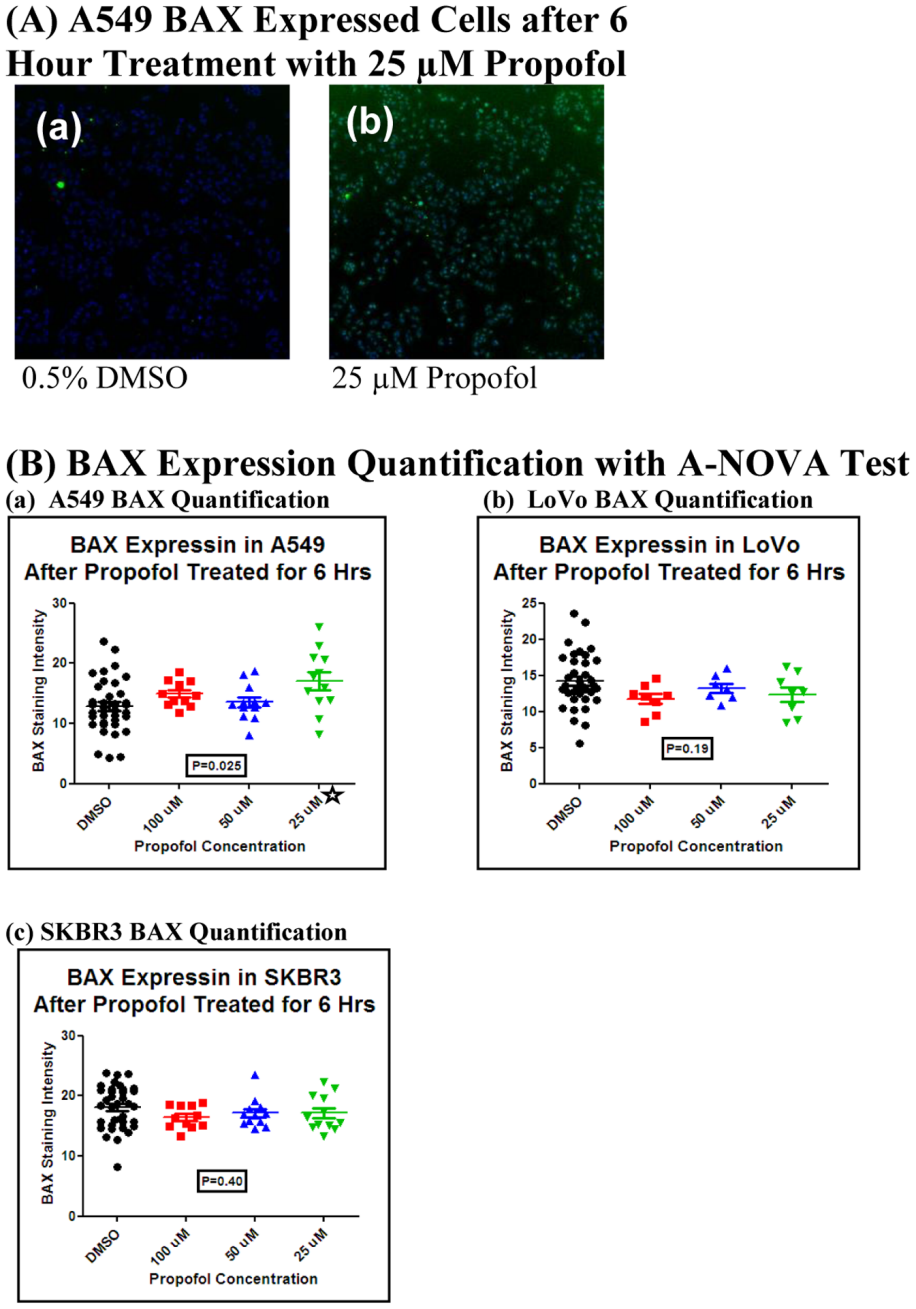
High Content Analysis of BAX expression in three cell lines. Propofol treatment increased the BAX expression in A549 cells treated for 6 hours with 25 µM of propofol. No change in BAX expression in all other groups and other two cell lines. (A) High content analysis of expression BAX in A549 cell treated with 25 µM of propofol for 6 hour. Nuclei were stained blue. BAX positive cells were stained green. Images were acquired at 5× magnification lens. (a) A549 cell treated with 0.5% DMSO for 6 hours. (b) A549 treated with 25 µM of propofol made with 0.5% DMSO for 6 hours. These results are representative of three independent experiments. (B) Charts indicate BAX expression in A549 (a), LoVo (b), and SKBR3 (c) lines with the induction of propofol for 6 hours. There is no BAX signal change after 24 hour treatment with all three concentrations of propofol for any of the three lines tested here. Each value was determined with Target Activation BioApplication Guide suggested by manufacturer (Thermo Scientific), and normalized to the expression of BAX with 0.5% DMSO incubation, and represents a mean of triplicate determinants. The results were evaluated by ANOVA test. A star (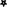) indicates statistically significant difference with the P<0.05.

To determine the effects of propofol on the induction of apoptosis for these three cell lines (A549, LoVo, and SKBR3), BAX was selected. BAX expression level was significantly high after a 6 hour treatment with 25 µM propofol in A549 cells (Fig. 2). There were, however, no changes in the 50 µM or 100 µM groups at either the 6 hour or 24 hour time points. Additionally, there was no change in BAX expression in either LoVo or SKBR3 cells treated with 25 µM, 50 µM or 100 µM of propofol.

### Phosphorylated ERK Expression Level Induced by Propofol in A549 Cells

**Figure 3:**
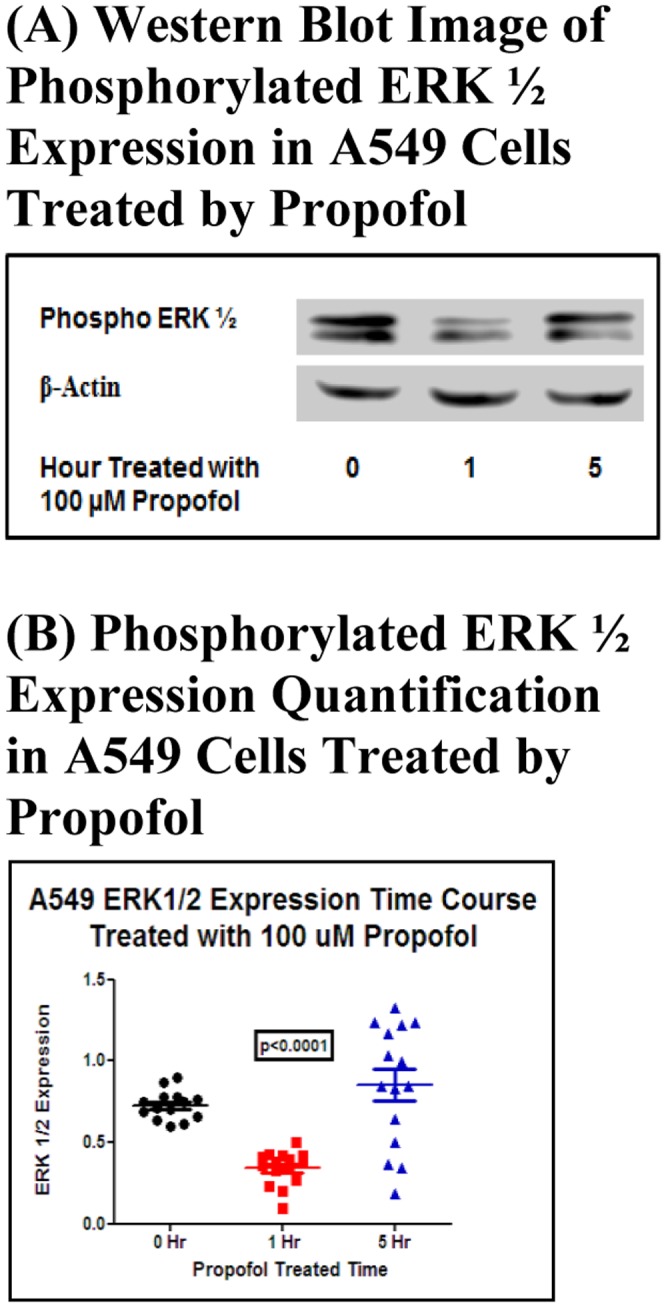
Propofol treatment decreased ERK1/2 activation in A549 cells. (A) The Western Blot Image of time course of Phosphorylated ERK1/2 Expression in A549 Treated with 100 µM propofol. (B) The Quantification of the Western Blot. These results are representative of three independent experiments with a total of 15 samples. By using One Way ANOVA testing, we found the differences from all one-hour propofol groups to be significantly lower compared with the 0-hour group with a P value <0.0001. ANOVA was used to do the analysis.

ERK is one of the key factors in the Ras/Raf/MEK/ERK signaling pathway. This pathway promotes cell proliferation, cell survival and metastasis. Usually it is aberrantly activated in cancer [17]. The activation of ERK is associated with K-Ras mutation in cancer cells [18]). We next sought to determine the activation of ERK1/2 in K-Ras mutant A549 cells after propofol treatment. We found that the phosphorylated ERK1/2, the activated form of ERK1/2, started to decrease at 60 minutes after propofol treatment and returned to the basal level at 5 hour time point (Fig. 3).

### Bioinformatics data search

To explain the caspase3 expression difference among these three tested cell lines, the Cancer Cell Line Encyclopedia (CCLE) database was searched. We found that both A549 and LoVo cells carry mutations of Kras at codon 12 and 13, respectively, while the SKBR3 cells have no such mutations. (Table1). Among the 177 Kras mutations, the missense mutations at codon 12 and 13 are the most frequent (73%) (Table2), indicating the importance of these two codons in tumor genesis and resistance to treatment.

**Table 1:** Results of Propofol Induced Apoptosis in Cancer Cells with Different Kras Gene Types.

Cell	KRAS Protein Change	Chromosome	Variant Classification	Propofol Induced Apoptosis
A549 LUNG	p.G12S	12	Missense Mutation	Induced
LoVo LARGE INTESTINE	p.G13D	12	Missense Mutation	Trend to be Induced
MDAMB231 BREAST	p.G13D	12	Missense Mutation	Induced
MIAPACA2 PANCREAS	p.G12C	12	Missense Mutation	Induced
HepG2		12	UTR 5	Induced
SKBR3			Wild Type	Not Induced
SH-SY5Y			Wild Type	Not Induced
SNU 761			Wild Type	Not Induced

**Table 2:** Kras Mutation Statistics.

Mutation Position	Mutation Number	% of Total
Kras Mutation at Codon 12	115	65
Kras Mutation at Codon 13	15	8
Kras Mutation at Codon 61	10	6
Kras Mutation at Codon 146	5	3
Kras Mutation at Codon 59	3	2
Kras Mutation at Codon 14	3	2
Kras Mutation at Other Codons	26	14
Total Kras Mutations	177	100

## Conclusions

Propofol is extensively used as a sedative and general anesthetic for surgery. Additionally, recent studies demonstrated that propofol exerts anti-cancer effects by inducing cancer cell apoptosis [5], [7], [19]. Propofol may directly and indirectly suppress the viability and proliferation of cancer cells by promoting apoptosis in human promyelocytic leukemia HL-60 and hepatic cancer cells (HepG2) [10], [20]. Siddiqui *et al* reported that low propofol concentrations (10–100 µM) produced minimal caspase-3 expression in breast cancer cells (MDA-MB-231), while this effect was more profound with significant increases in caspase-3 when propofol was conjugated with omega-3 polyunsaturated long-chain fatty acids [7]. Qi-Hang Du *et al* revealed that treating pancreatic cancer cells (MIA-PaCa-2) with propofol in concentrations of 10, 25, 50, and 100 µmol/mL resulted in dose- and time-dependent promotions of apoptosis. They concluded that propofol was a single effective promoter of the death of pancreatic cancer cells [21]. However, inconclusive results from other studies suggest variability in the biological behavior of propofol, implying a possible correlation between propofol and cancer promotion [22].

One of the biggest hurdles of cancer therapy is the heterogeneous genome of cancer cells. Different cancer cells with various oncogenes may respond differently to propofol treatment [7], [21], [22], [23]. A very common mutation in the EGFR signaling pathway in cancer is the Kras mutation [23], [24], [25]. We extended the scope of our cancer cell model by specifically focusing on the Kras mutations status. Two Kras mutant cell lines, a non-small cell lung carcinoma A549 and a human colon carcinoma LoVo, and a Kras wild-type human breast cancer SK-BR-3 cell line were selected in our study to establish a mini profile for quantitative and functional evaluation of propofol-induced apoptosis. We understand that signaling pathways are activated independently of EGFR when the Kras gene is mutated, which leads to continual signal transduction and stimulation of downstream signaling pathways involved in cell growth, proliferation, invasion, and metastasis [23]. In our study, caspase-3 had been used as an apoptotic bio-surrogate to detect apoptosis. Our data showed that propofol-induced apoptosis in A549 cells with high caspase-3 expression occurs in a dose-dependent manner and significantly rises at the concentration of 100 µM propofol treatment for 24 hours. However, there was no increase of caspase-3 expression in SK-BR-3 cells. We noted a trend toward an increased expression of caspase-3 associated with the increased concentration of propofol treatment from 25 µM to 100 µM in LoVo, although there was no statistically significant difference in the apoptotic process after 24-hour induction in LoVo cells.

BAX, a Bcl 2 family member involved in the mitochondria-dependent pathway [26], [27], was employed in our study as another bio-surrogate. Our data showed highly expressed BAX in A549 cells treated with 25 µM of propofol for 6 hours. The time of more expression of Bax at 6 hours, which was earlier than caspase-3 expression at 24 hours in our study, is consistent with the natural process of apoptosis. There was no change in BAX expression in all three propofol concentration groups in LoVo cells and SKBR3 cells at both the 6- and 24- hour time points compared with the control groups.

The EGFR signaling pathway is regulated by a series of proteins and phosphatases and is associated with cell proliferation and growth. ERK1/2 is an important mitogen-activated protein kinase that controls several cellular activities and physiological processes. Usually the activation of ERK1/2 promotes cell survival [28]. Much clinical and laboratory research suggests that phospho-ERK1/2 expression was dramatically down-regulated while these MAPK inhibitors were applied, and tumor cell growth was effectively controlled [29], [30]. In our study, the inhibition of phospho-ERK1/2 was observed in A549 cells after a 1-hour treatment with 100 µM propofol and returned to the basal level after 5-hour treatment with 100 µM propofol. This finding suggests that propofol may target one of the steps in the EGFR signaling pathway and induce apoptosis in A549 cells.

Interestingly, we observed that cancer cells with the Kras mutation were susceptible or had a tendency to be susceptible to propofol treatment, while cancer cells with wild-type Kras lacked such response. To confirm the findings and explore whether the Kras mutation at different codons may affect the behavior of cancer cells differently with propofol treatment, we employed bioinformatics data (Tables 1 and 2) from the CCLE database, and then compared our results with published data (7, 21). We realized that MDAMB231, HepG2, and MiaPack2 all carry the Kras mutation. Both A549 and MiaPack2 are mutated at codon 12 of Kras [31], whereas both LoVo and MDAMB231 are mutated at codon 13 [32] (Table 1). On the other hand, Kras wild type cancer cells failed to undergo induced apoptosis, when treated with propofol and demonstrated a protective response from oxidative stress related apoptosis [33], [34]. This may explain the different cell behaviors in response to propofol treatment among these types of cancer cell.

There is a limitation to this study: although BAX was highly expressed in A549 cells treated with 25 µM propofol for 6 hours, BAX expression was not significant when the cells treated with 50 and 100 µM propofol. Further studies are required to better elucidate these findings.

Our study provides new insights into the effects of propofol on the behavior of different cancer cells. We consider that the Kras mutation status of cancer cells may be a potential indicator and worthy of evaluation with a large-scale screening of propofol induction of apoptosis. Because the Kras codon 12 mutation is the most common mutation with 65% of total Kras mutations in cancer cells, while the codon 13 mutation is approximately 8% (Table 2), cancer cells with the Kras codon 12 mutation should be further considered as a screening target to confirm the relationship between the Kras genotype and the propofol effect on them. This would, in turn, provide more accurate information for precision medicine development, in so doing, tailor the treatment to the genetic profile of each cancer patient.

## References

[1] Mammotoa T, Mukaib M, Mammotoc A, Yamanakab Y, Hayashid Y, et al (2002) Intravenous anesthetic, propofol inhibits invasion of cancer cells. Cancer Letters 184:165–170. 12127688 10.1016/s0304-3835(02)00210-0

[2] Ren XF, Li WZ, Meng FY, Lin CF (2010) Differential effects of propofol and isoflurane on the activation of T-helper cells in lung cancer patients. Anesthesia 65:478–482. 10.1111/j.1365-2044.2010.06304.x 20337621

[3] Huang H, Benzonana L, Zhao H, Watts H, Perry N, Bevan C, et al (2014) Prostate cancer cell malignancy via modulation of HIF-1α pathway with isoflurane and propofol alone and in combination. British Journal of Cancer. 111:1338–1349. 25072260 10.1038/bjc.2014.426 PMC4183852

[4] Homburger J, Meiler S (2006) Anesthesia drugs, immunity, and long-term outcome. Current Opinion in Anaesthesiology 19:423–428. 16829725 10.1097/01.aco.0000236143.61593.14

[5] Melamed R, Bar-Yosef S, Shakhar G, Ben-Eliyahu S (2003) Suppression of natural killer cell activity and promotion of tumor metastasis by ketamine, thiopental, and halothane, but not by propofol: mediating mechanisms and prophylactic measures. Anesth Analg 97:1331–1339. 14570648 10.1213/01.ANE.0000082995.44040.07

[6] Wada H, Seki S, Takahashi T, Kawarabayashi N, Higuchi H, et al (2007) Combined spinal and general anesthesia attenuates liver metastasis by preserving TH1/TH2 cytokine balance. Anesthesiology 106:499–506. 17325508 10.1097/00000542-200703000-00014

[7] Siddiqui RA, Zerouga M, Wu M, Castillo A, Harvey K, et al (2005) Anticancer properties of propofol-docosahexaenoate and propofol-eicosapentaenoate on breast cancer cells Breast Cancer Research. 7:645–654. 10.1186/bcr1036 PMC1242121 16168109

[8] de Visser KE, Eichten A, Coussens LM (2006) Paradoxical roles of the immune system during cancer development. Nature Review Cancer 6:24–37. 16397525 10.1038/nrc1782

[9] Miao Y, Zhang Y, Wan H, Chen L, Wang F (2010) GABA-receptor agonist, propofol inhibits invasion of colon carcinoma cells. Biomedicine & Pharmacotherapy 64:583–588. 20888181 10.1016/j.biopha.2010.03.006

[10] Tsuchiya M, Asada A, Arita K, Utsumi T, Yoshida T, et al (2002) Induction and mechanism of apoptotic cell death by propofol in HL-60 cells. Acta Anaesthesiol Scand 46:1068–1074. 12366500 10.1034/j.1399-6576.2002.460903.x

[11] Xu YB, Du QH, Zhang MY, Yun P, He CY (2013) Propofol suppresses proliferation, invasion and angiogenesis by down-regulating ERK-VEGF/MMP-9 signaling in Eca-109 esophageal squamous cell carcinoma cells. European Review for Medical and Pharmacological Sciences 17:2486–2494. 24089228

[12] Chiu WT, Lin YL, Chou CW, Chen RM (2009) Propofol inhibits lipoteichoic acid-induced *iNOS* gene expression in macrophages possibly through down regulation of toll-like receptor 2-mediated activation of Raf-MEK1/2-ERK1/2-IKK-NFκB. Chemico-Biological Interactions. 181:430–439. 19573522 10.1016/j.cbi.2009.06.011

[13] Duldulao MP, Lee W, Nelson RA, Li W, Chen Z, et al (2013) Mutations in specific codons of the KRAS oncogene are associated with variable resistance to neoadjuvant chemoradiation therapy in patients with rectal adenocarcinoma. Ann Surg Oncol. 20:2166–2171. 23456389 10.1245/s10434-013-2910-0 PMC5584556

[14] Bennetts PS, Pierce JD (2010) Apoptosis: Understanding Programmed Cell Death for the CRNA. AANA Journal 78:237–245. 20572411

[15] Steelman LS, Franklin RA, Abrams SL, Chappell W, Kempf CR, et al (2011) Roles of the Ras/Raf/MEK/ERK pathway in leukemia therapy. Leukemia 25:1080–1094. 21494257 10.1038/leu.2011.66

[16] Chappell WH, Steelman LS, Long JM, Kempf RC, Abrams SL, et al (2011) Ras/Raf/MEK/ERK and PI3K/PTEN/Akt/mTOR Inhibitors: Rationale and Importance to Inhibiting These Pathways in Human Health. Oncotarget 2:135–164. 21411864 10.18632/oncotarget.240 PMC3260807

[17] Roberts PJ, Der CJ (2007) Targeting the Raf-MEK-ERK mitogen-activated protein kinase cascade for the treatment of cancer. Oncogene fet 26:3291–3310. 10.1038/sj.onc.1210422 17496923

[18] Schmitz KJ, Wohlschlaeger J, Alakus H, Bohr J, Stauder MA, et al (2007) Activation of extracellular regulated kinases (ERK1/2) but not AKT predicts poor prognosis in colorectal carcinoma and is associated with k-ras mutations. Virchows Arch 450:151–159. 17149612 10.1007/s00428-006-0342-y

[19] Kushida A, Inada T, Shingu K (2007) Enhancement of Antitumor Immunity after Propofol Treatment in Mice. Immunopharmacology and Immunotoxicology 29:477–486. 18075859 10.1080/08923970701675085

[20] Zhang J, Wu GQ, Zhang Y, Feng ZY, Zhu SM (2013) Propofol induces apoptosis of hepatocellular carcinoma cells by up regulation of microRNA-199a expression. Cell Biol Int. 37:227–232. 23319430 10.1002/cbin.10034

[21] Du QH, Xu YB, Zhang MY, Yun P, He CY (2013) Propofol induces apoptosis and increases gemcitabine sensitivity in pancreatic cancer cells *in vitro* by inhibition of nuclear factor-κB activity. World J Gastroenterol. 19:5485–5492. 24023491 10.3748/wjg.v19.i33.5485 PMC3761101

[22] Zhang L, Wang N, Zhou S, Ye W, Jing G, et al (2012) Propofol induces proliferation and invasion of gallbladder cancer cells through activation of Nrf2. Journal of Experimental & Clinical Cancer Research 31:66. 22901367 10.1186/1756-9966-31-66 PMC3502506

[23] Riely GJ, Marks J, Pao W (2009) KRAS Mutations in Non–Small Cell Lung Cancer. Proc Am Thorac Soc 6:201–205. 19349489 10.1513/pats.200809-107LC

[24] Boch C, Kollmeier J, Roth A, Stephan-Falkenau S, Misch D, et al (2013) The frequency of EGFR and KRAS mutations in non-small cell lung cancer (NSCLC): routine screening data for central Europe from a cohort study. BMJ Open 3:1–7. 10.1136/bmjopen-2013-002560 PMC3641502 23558737

[25] Takamochi K, Oh S, Suzuki K (2013) Differences in EGFR and KRAS mutation spectra in lung adenocarcinoma of never and heavy smokers. ONCOLOGY LETTERS 6:1207–1212. 24179496 10.3892/ol.2013.1551 PMC3813793

[26] Lalier L, Pedelaborde F, Braud C, Menanteau J, Vallette FM, et al (2011) Increase in intracellular PGE2 induces apoptosis in Bax-expressing colon cancer cell BMC Cancer. 11:153. 10.1186/1471-2407-11-153 PMC3097003 21524287

[27] Gibson LF, Fortney J, Magro G, Ericson SG, Lynch JP, et al (1999) Regulation of BAX and BCL-2 expression in breast cancer cells by chemotherapy. Breast Cancer Research and Treatment 55:107–117. 10481938 10.1023/a:1006175811676

[28] Lu Z, Xu S (2006) ERK1/2 MAP Kinases in Cell Survival and Apoptosis. IUBMB Life 58:621–631. 17085381 10.1080/15216540600957438

[29] Chappell WH, Steelman LS, Long JM, Kempf RC, Abrams SL, et al (2011) Ras/Raf/MEK/ERK and PI3K/PTEN/Akt/mTOR Inhibitors: Rationale and Importance to Inhibiting These Pathways in Human Health. Oncotarget 2:135–164. 21411864 10.18632/oncotarget.240 PMC3260807

[30] Liu L, Cao Y, Chen C (2006) Sorafenib Blocks the RAF/MEK/ERK Pathway, Inhibits Tumor Angiogenesis, and Induces Tumor Cell Apoptosis in Hepatocellular Carcinoma Model PLC/PRF/5. Cancer Res 66:11851–11858. 17178882 10.1158/0008-5472.CAN-06-1377

[31] Singh A, Greninger P, Rhodes D, Koopman L, Violette S, et al (2009) A Gene Expression Signature Associated with “K-Ras Addiction” Reveals Regulators of EMT and Tumor Cell Survival. Cancer Cell 15:489–500. 19477428 10.1016/j.ccr.2009.03.022 PMC2743093

[32] Vachtenheim J, Horáková I, Novotná H (1995) Mutations of K-ras oncogene and absence of H-ras mutations in squamous cell carcinomas of the lung. Clin Cancer Res 1:359–365. 9815992

[33] Gu J, Chi M, Sun X, Wang G, Li M, et al (2013) Propofol-Induced Protection of SH-SY5Y Cells against Hydrogen Peroxide Is Associated with the HO-1 via the ERK Pathway. Int J Med Sci 10:599–606. 23569422 10.7150/ijms.5151 PMC3619098

[34] Lee JY, Shin JW, Lee EH, Baek SH, Ku SW, et al (2010) Comparison of the effects of propofol and pentobarbital on hydrogen peroxide-stimulated hepatic SNU761 cells. Korean J Anesthesiol. 58:277–82. 20498778 10.4097/kjae.2010.58.3.277 PMC2872837

